# Actinomyces israelii Brain Abscess With Advanced HIV Disease

**DOI:** 10.7759/cureus.79544

**Published:** 2025-02-24

**Authors:** Laura Leite-Almeida, Maria Sousa, Teresa Magalhães, Sandra Rebelo, Josué Pereira, Ana Reis Melo, Margarida Tavares

**Affiliations:** 1 Pediatrics, Unidade Local de Saúde de São João, Porto, PRT; 2 Faculty of Medicine, University of Porto, Porto, PRT; 3 Pediatrics, Hospital do Divino Espírito Santo de Ponta Delgada, Ponta Delgada, PRT; 4 Pediatrics, Centro Hospitalar do Oeste, Caldas da Rainha, PRT; 5 Clinical Pathology, Unidade Local de Saúde de São João, Porto, PRT; 6 Neurosurgery, Unidade Local de Saúde de São João, Porto, PRT; 7 Pediatric Infectious Diseases, Unidade Local de Saúde de São João, Porto, PRT

**Keywords:** actinomyces israelii, bacterial cns infection, brain abscess, combination antiretroviral therapy, pediatric hiv infection

## Abstract

We present the first reported case of a pediatric central nervous system (CNS) abscess caused by *Actinomyces israelii* in the context of advanced HIV disease. A three-year-old girl from São Tomé and Príncipe presented with progressive neurological deficits, including gait instability and language delay. Brain MRI revealed a right temporal lobulated lesion with surrounding edema and mass effect. Chronic superior sagittal sinus thrombosis and hydrocephalus were also identified. Neurosurgical intervention included ventriculocisternostomy and microsurgical resection of the lesion, which revealed a multiloculated abscess. Histology confirmed granuloma formation, and polymerase chain reaction (PCR) identified *A. israelii*. HIV serology was positive, with a CD4 count of 664 cells/μL and a viral load of 1,340,000 copies/mL. The patient received a year-long antibiotic regimen, starting with intravenous penicillin G followed by oral amoxicillin, and antiretroviral therapy was initiated. She showed marked improvement in neurological function and no signs of relapse after one year. This case underscores the importance of considering *A. israelii* in the differential diagnosis of CNS lesions in immunocompromised pediatric patients. It also highlights the critical role of neurosurgery, molecular diagnostics, and multidisciplinary management in ensuring favorable outcomes.

## Introduction

Actinomyces species are Gram-positive, facultative anaerobic, filamentous bacteria that are part of the normal flora of the human oral cavity, gastrointestinal tract, and female genital tract [1,2]. *Actinomyces israelii*, the most commonly isolated species, is an opportunistic pathogen that can cause actinomycosis, which is a rare, chronic disease characterized by abscess formation, tissue fibrosis, and sinus tract development [2,3]. While cervicofacial actinomycosis is the most frequent presentation, *A. israelii* can also cause thoracic, abdominal, and central nervous system (CNS) infections, particularly in immunocompromised hosts [1,2]. CNS involvement in actinomycosis is rare, accounting for less than 2% of all reported cases [4].

We present the first reported case of a pediatric CNS abscess caused by *A. israelii* in the context of advanced HIV disease.

This case was previously presented at the 42nd Annual Meeting of the European Society for Paediatric Infectious Diseases, held from May 20 to 24, 2024.

## Case presentation

A three-year-old girl from São Tomé and Príncipe presented with progressive neurological deficits. One year earlier, she had been hospitalized in her home country for suspected meningoencephalitis. At that time, she exhibited fever, malaise, vomiting, headaches, neck hyperextension, and amaurosis. On neurological examination, she had a Glasgow Coma Scale score of 13, positive Kernig and Brudzinski signs, and absent pupillary reflexes. Due to concerns of intracranial hypertension and the unavailability of neuroimaging, a lumbar puncture was not performed, and empirical treatment was initiated with ceftriaxone, vancomycin, and acyclovir. She completed a 10-day course of ceftriaxone and vancomycin and a 14-day course of acyclovir, with some clinical improvement.

Following her initial hospitalization, her condition deteriorated, resulting in a complete loss of ambulation and speech. However, her father reported a spontaneous and gradual recovery over several months, with partial improvements in ambulation and speech. Due to limited access to imaging resources, she was transferred to Portugal for further evaluation.

**Figure 1:**
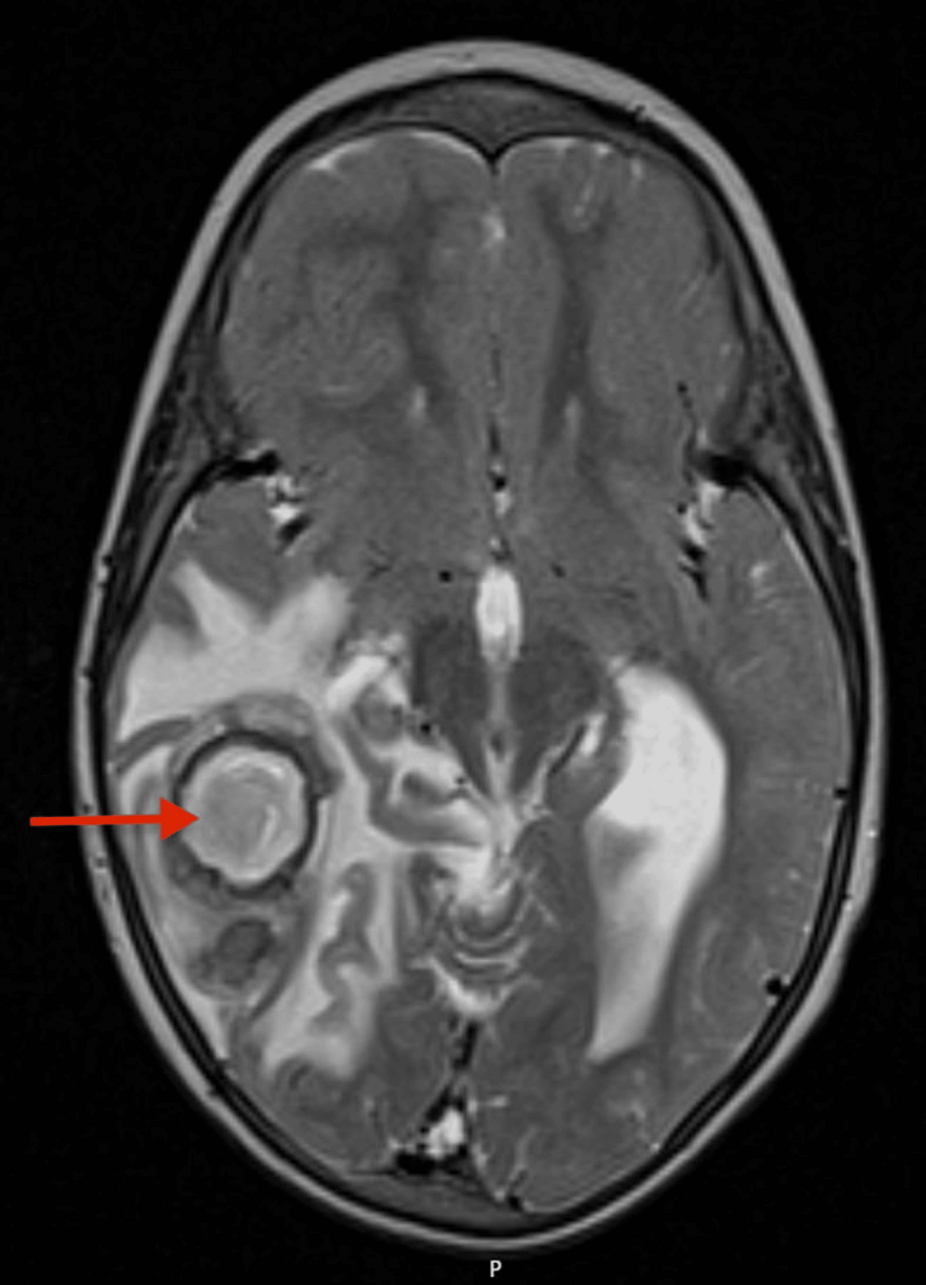
Axial T2-weighted MRI shows a large, well-defined hyperintense right temporal lesion with surrounding vasogenic edema and midline shift

In Portugal, she presented with gait instability, frequent falls, and language delay. Neurological examination showed brisk reflexes, clonus, a positive Babinski sign on the right, and asymmetric lower limb movements. Her gait was wide-based and unsteady. Initial brain magnetic resonance imaging (MRI) revealed a right temporal lobulated lesion measuring 4.2 × 3 × 2.8 cm with peripheral, thick, multilobulated contrast enhancement. Extensive surrounding vasogenic edema caused mass effect, with midline shift (Figure 1). Additional findings included chronic superior sagittal sinus thrombosis and hydrocephalus.

Laboratory tests showed microcytic anemia and mildly elevated inflammatory markers (C-reactive protein, 4.5 mg/L; erythrocyte sedimentation rate, 38 mm/h) without other significant findings. Thoracoabdominal computed tomography revealed generalized lymphadenopathy. Latent tuberculosis was ruled out using an interferon-gamma release assay. HIV serology was positive, with a CD4 count of 664 cells/μL (16.6%) and a viral load of 1,340,000 copies/mL, consistent with stage 2 of the Centers for Disease Control and Prevention classification [5]. Next-generation sequencing HIV resistance testing revealed a K103N mutation. Blood cultures were negative.

**Figure 2:**
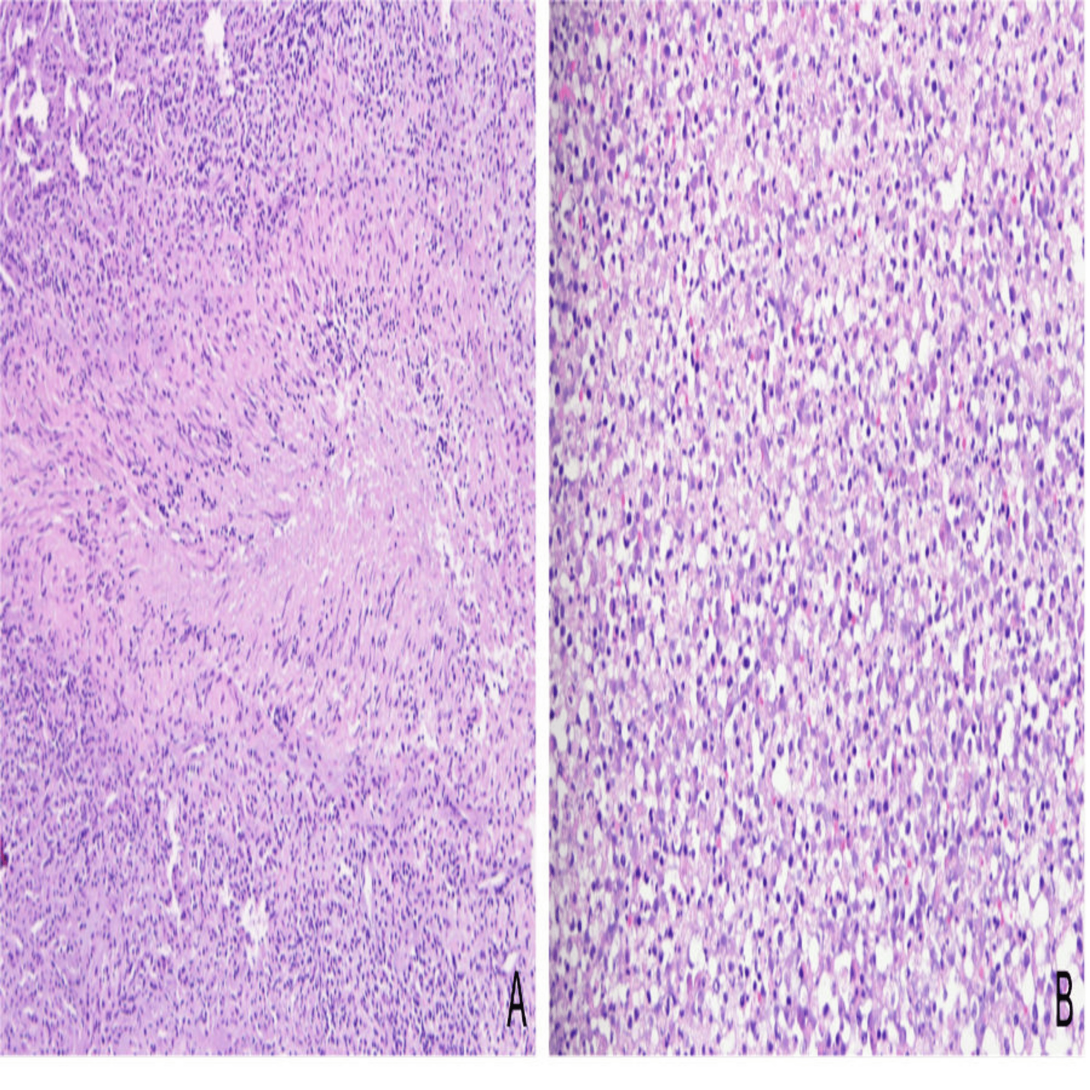
Histological examination disclosed a chronic and acute inflammatory process disclosing epithelioid granuloma with central necrosis (A: H&E, 100x) and foci of suppurative inflammation (B: H&E, 200x)

Neurosurgical interventions on day 5 of admission included an endoscopic third ventriculocisternostomy and microsurgical resection of the entire temporal lesion. Intraoperatively, a hemorrhagic, thick encapsulated, multiloculated lesion with milky purulent drainage was identified. Meropenem was started immediately. Histological analysis revealed chronic inflammatory changes with granuloma formation (Figure 2). Molecular testing using polymerase chain reaction (PCR) targeting the 16S rRNA gene was positive for *Actinomyces israelii* and negative for other microorganisms tested (*Mycobacterium tuberculosis* complex, *M. avium*, *Toxoplasma gondii*, *Treponema pallidum*, *Nocardia*, *Listeria*, *Cryptococcus*, *Histoplasma capsulatum*, *Aspergillus fumigatus*, *Candida albicans*, JC virus, Epstein-Barr virus, and cytomegalovirus). Microbiological analyses of the pus, including bacteriological, mycobacterial, and fungal cultures, were negative.

**Figure 3:**
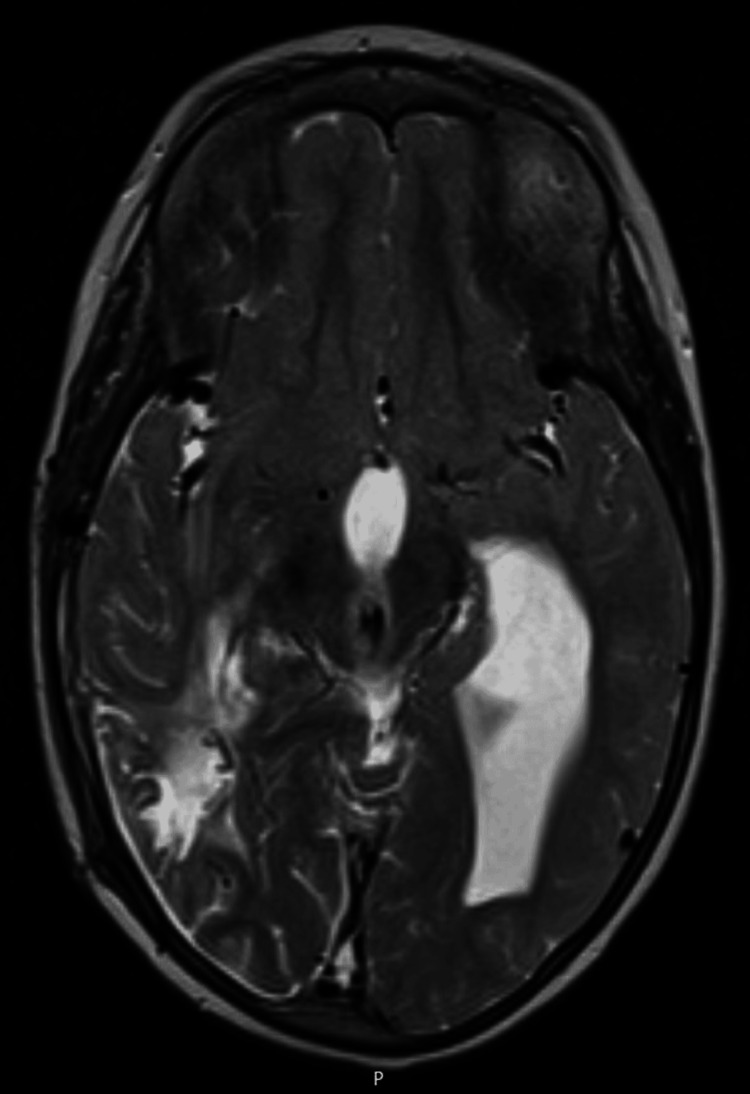
Axial T2-weighted post-surgical MRI

Following molecular confirmation of *Actinomyces israelii*, antibiotics were switched to intravenous penicillin G, followed by high-dose oral amoxicillin for one year. Antiretroviral therapy (ART) with raltegravir, lamivudine, and abacavir was initiated, since pediatric formulation of dolutegravir was unavailable in Portugal. During her hospitalization, the patient participated in a motor rehabilitation program, showing marked improvement in gait and motor function. Post-surgery brain MRI (Figure 3) showed progressive improvement, and follow-up imaging at 3, 6, and 12 months did not show any radiological findings of relapse. Three months after initiating ART, the viral load became undetectable, and the CD4 count was 1408 cells/μL (38%). She showed ongoing clinical improvement, with stable neurological function, no amaurosis, and no signs of relapse.

## Discussion

CNS actinomycosis poses significant diagnostic challenges, due to nonspecific clinical and radiological features, mimicking malignancies, tuberculosis, or nocardiosis [3,4]. In our case, MRI suggested an indolent infectious process, but definitive diagnosis relied on PCR, which identified *A. israelii*. This underscores the importance of molecular diagnostics when conventional culture methods fail, either due to prior antibiotic use or the fastidious nature of the organism [1,4]. Molecular testing provides a rapid and accurate diagnosis, enabling the initiation of targeted therapy and avoiding unnecessary delays that could lead to further complications [2,4].

CNS actinomycosis requires prolonged antibiotic therapy, with several months to ensure complete eradication and prevent relapse. Penicillin remains the cornerstone of treatment, typically initiated intravenously, followed by an extended course of oral antibiotics [2,4,6]. Surgical intervention is frequently necessary, as was the case for our patient, to drain abscesses and alleviate mass effects, especially when significant neurological compromise is present [6,7]. Early and aggressive treatment, combining surgical and medical approaches, is critical to improving outcomes, particularly in immunocompromised individuals [6,7].

Advanced HIV disease in our patient likely contributed to the progression to CNS actinomycosis. Advanced HIV disease (AHD) is defined by severe immunosuppression and heightened susceptibility to opportunistic infections and mortality. According to World Health Organization (WHO) guidelines, all children under five are classified as having AHD, reflecting their increased vulnerability [8,9]. Younger children often present with advanced immunosuppression, making them more susceptible to severe and atypical infections, such as this one. The WHO's STOP AIDS framework (Screen-Treat-Optimize-Prevent) emphasizes early diagnosis, timely ART, and preventive strategies to reduce morbidity and mortality [8,9]. In our patient, prompt initiation of ART stabilized her immune function, resulting in viral suppression and immune recovery, as evidenced by her improving CD4 count and low viral load. This case highlights the critical importance of early HIV diagnosis and management, particularly in children presenting with atypical infections.

## Conclusions

This case underscores the importance of considering *Actinomyces israelii* in the differential diagnosis of indolent CNS lesions, particularly in immunocompromised individuals. The concurrent diagnosis of CNS actinomycosis and HIV is exceptionally rare, and, to the best of our knowledge, this represents the first documented case in a child. It emphasizes the importance of advanced molecular diagnostics and early, aggressive management combining surgery, prolonged antibiotics, and ART to optimize outcomes.
